# The temporal expression profile of a Nos3-related natural antisense RNA in the brain suggests a possible role in neurogenesis

**DOI:** 10.1016/j.niox.2017.10.002

**Published:** 2017-12-01

**Authors:** Gabriela G. Chavez, Gabriella Taylor, Jekaterina Garaliene, Guy P. Richardson, Sergei A. Korneev

**Affiliations:** Sussex Neuroscience, School of Life Science, University of Sussex, Brighton, BN1 9QG, UK

**Keywords:** Nos, Nitric Oxide Synthase, NAT, Natural Antisense Transcript

## Abstract

Experimental work over the past several years has revealed an unexpected abundance of long natural antisense transcripts (NATs) in eukaryotic species. In light of the proposed role of such RNA molecules in the regulation of gene expression in the brain, attention is now focused on specific examples of neuronal NATs. Of particular interest are NATs that are complementary to mRNAs encoding nitric oxide synthase (NOS), the enzyme responsible for production of the important gaseous neurotransmitter nitric oxide (NO). Here we study the temporal expression profile of murine *Nos3as* NAT in the brain. Notably, *Nos3as* NAT is known to act as a negative regulator of *Nos3* gene expression. The results of our quantitative analysis reveal differential expression of *Nos3as* NAT during embryonic and post-embryonic stages of development of the brain. Also, they show that the low levels of *Nos3as* NAT coincides with active neurogenesis. In addition we report on an inverse correlation between the relative expression level of *Nos3as* NAT and the level of Nos3 protein. Thus our data raise the hypothesis that the *Nos3as* NAT regulates neurogenesis through suppression of *Nos3* gene activity. This idea is further supported by experiments conducted on the olfactory bulbs and cultured neuroblastoma cells.

## Highlights

• *Nos3as* NAT and *Nos3* mRNA are differentially expressed during mouse brain development and aging.
• Low levels of *Nos3as* NAT coincides with active neurogenesis.
• There is an inverse correlation between the expression level of *Nos3as* NAT and the level of Nos3 protein in the brain.
• Neuronal differentiation of cultured neuroblastoma cells is associated with up-regulation of *Nos3as* NAT.
• *Nos3as* NAT may be involved in controlling both embryonic and adult neurogenesis.

## 1 Introduction

Long natural antisense transcripts (NATs) are endogenous RNA molecules that are complementary to RNA transcripts of already established function. They are longer than 200 nt and depending on their origin can be classified as *cis*-encoded and *trans*-encoded NATs. *Cis*-encoded NATs are produced from the same loci as their sense counterparts whereas *trans*-encoded NATs are transcribed from different loci. Recently, the cumulative efforts of molecular biologists and bioinformaticians have led to the discovery of many long NATs in eukaryotic systems [1], [2]. Probably the most exciting outcome of these studies is that long NATs appear to be especially prevalent in the nervous system [3]. One important class of long NATs that has been studied in both vertebrates and invertebrates contains RNA molecules involved in regulating the production of the endogenous gaseous neurotransmitter nitric oxide (NO) [4], [5], [6], [7]. In mammals, NO has been implicated in many physiological processes including the regulation of blood flow, neurotransmission, platelet aggregation and the immune response [8], [9], [10]. It is produced by three major nitric oxide synthase (NOS) isoforms: neuronal NOS (NOS1), inducible NOS (NOS2) and endothelial NOS (NOS3). In the brain, these isoforms are expressed either constitutively (NOS 1 and NOS3) or inducibly (NOS2). Notably, long NATs complementary to mRNAs encoding all three mammalian NOS isoforms have been reported in recent literature [5], [6], [11], [12]. Rodent *Mm-antiNos1* and *Nos3as* RNAs belong to a class of *cis*-encoded NATs and function as negative regulators of the *Nos1* and *Nos3* genes respectively [12], [13]. *Anti-NOS2a* is an example of a *trans*-encoded NAT and is likely to be involved in the regulation of *NOS2* gene in humans [6]. However, while *Mm-antiNos1*, *Nos3as* and anti-NOS2a NATs were found to be present in the nervous system, the temporal expression profile in the brain has been reported only for the *Mm-antiNos1* RNA [12]. In this study we report on the results of a quantitative RNA analysis that reveals differential expression of *Nos3as* RNA during embryonic and post-embryonic stages of brain development. We demonstrate an inverse correlation between the relative expression level of *Nos3as* RNA and the level of Nos3 protein produced in the brain. We also show that in the olfactory bulb (OB) *Nos3as* RNA expression remains very low in both embryonic and perinatal mice. In addition, we report that neuronal differentiation of murine neuroblastoma Neuro2a cells is associated with up-regulation of *Nos3as* RNA. Taken together our data suggest that *Nos3as* RNA can be involved in the regulation of neurogenesis.

## 2 Methods

### 2.1 Animal use and tissue collection

Mice (CD1) and mouse embryos were killed by decapitation or cervical dislocation in accordance with Home Office guidelines and either the whole brain or the olfactory bulb were rapidly dissected in ice cold PBS and frozen in liquid nitrogen to minimise RNA degradation. All procedures were performed with the approval of the University of Sussex Animal Welfare and Ethical Review Committee and in accordance with UK Home Office regulations.

### 2.2 Maintenance and differentiation of Neuro2a cells

Mouse Neuro2a cells were obtained from the American Type Culture Collection. Cells were cultured in DMEM (Gibco) containing 10% heat-inactivated FCS (PAN Biotech) and penicillin/streptomycin (Gibco) at 37 °C in 95% air, 5% CO2. For differentiation assays, Neuro2a cells were plated into 100 mm Petri dishes to reach 25% confluence. The cells were then differentiated by incubation in DMEM containing reduced serum medium (0.1% FCS) and 30 μM retinoic acid (Sigma). After 4 days the cells were washed with PBS and collected for further analysis.

### 2.3 Real-time reverse transcription-PCR

Total RNAs were extracted from individual samples of brain tissue (n = at least 4 animals per group) or Neuro2a cultured cells by means of the Absolutely RNA Microprep or Miniprep kits (Agilent Technologies). To remove all traces of genomic DNA the extracted RNAs were treated with DNase TURBO (Ambion). Purified RNAs were copied into cDNAs using the iScript cDNA synthesis kit (Bio-Rad). cDNAs were amplified and analyzed on a Mx3000P real-time cycler (Stratagene) using the iTaq Universal SYBR Green Supermix (Bio-Rad). We used primers 5′- AGAGAATTCTGGCAACAGAG-3 and 5′- TGGGTGCGCAATGTGAGT-3′ for detection of *Nos3* mRNA, primers 5′- GCCTCCACGCTATTTACC-3′ and 5′- TCCTCATCAGGTGAGCCT-3' for detection of *Nos3as* RNA and primers 5′-TGTCTCCTGCGACTTCAAC-3′ and 5′-AGCCGTATTCATTGTCATACC-3′ for detection of *GAPDH* mRNA. The identity of all PCR products was confirmed by sequencing. The amount of target transcript, normalized to an endogenous reference (*GAPDH*) and relative to a calibrator was calculated as 2^−ΔΔCt^ where ΔΔCT = ΔCT − ΔCT(CAL). ΔCT and ΔCT(CAL) are the differences in threshold cycles for target and reference (*GAPDH*) measured in the samples and in the calibrator (CAL) respectively. The ratio between *Nos3as* RNA and *Nos3* mRNA expression was calculated as 2^−ΔCt(Nos3as)^/2^−ΔCt(Nos3)^. The results of real-time RT-PCR were statistically evaluated by one-way ANOVA with post-hoc Tukey HSD test.

### 2.4 Western blotting

Brains were homogenized on ice in seven volumes of RIPA lysis buffer (Source BioScience) supplemented with phosphatase and protease inhibitor cocktails (Sigma-Aldrich). The homogenates were incubated for 1 h on ice and insoluble constituents were removed by centrifugation of the homogenates at 16,000 g and 4° C for 30 min. Supernatants were collected and protein quantification was performed using the Quick Start Bradford protein assay (Bio-Rad). Equal amounts of proteins were loaded on a TruPAGE 4–12% polyacrylamide gradient gel (Sigma-Aldrich) and run at slow voltage. Separated proteins were blotted onto Amersham Hybond-P PVDF membrane (GE Healthcare Life Sciences) using the Mini *Trans*-Blot electrophoretic transfer module (Bio-Rad). After protein transfer, the membrane was cut into two sections. The upper section contained proteins with a molecular weight greater than 50 kDa and the lower section contained proteins with a molecular weight below 50 kDa. Both sections were blocked with 5% non-fat dry milk in Tris-buffered saline (TBS) containing 0.15% Tween-20. The upper section was then incubated with rabbit polyclonal anti-Nos3 Ab-1177 antibodies (Sigma-Aldrich) at 1:500 and the lower section was incubated with rabbit polyclonal anti-GAPDH ABS16 antibodies (Millipore) at 0.25 μg/ml. HRP-conjugated goat anti-rabbit IgG at 1:2000 (Millipore) was used to detect the bound primary antibodies. Immunoreactive protein bands were detected and visualized using the Immobilon western chemiluminescent HRP substrate (Millipore).

## 3 Results

### 3.1 Quantitative RNA analysis reveals differential expression of *Nos3as* RNA during embryonic and post-embryonic stages of brain development

**Fig. 1:**
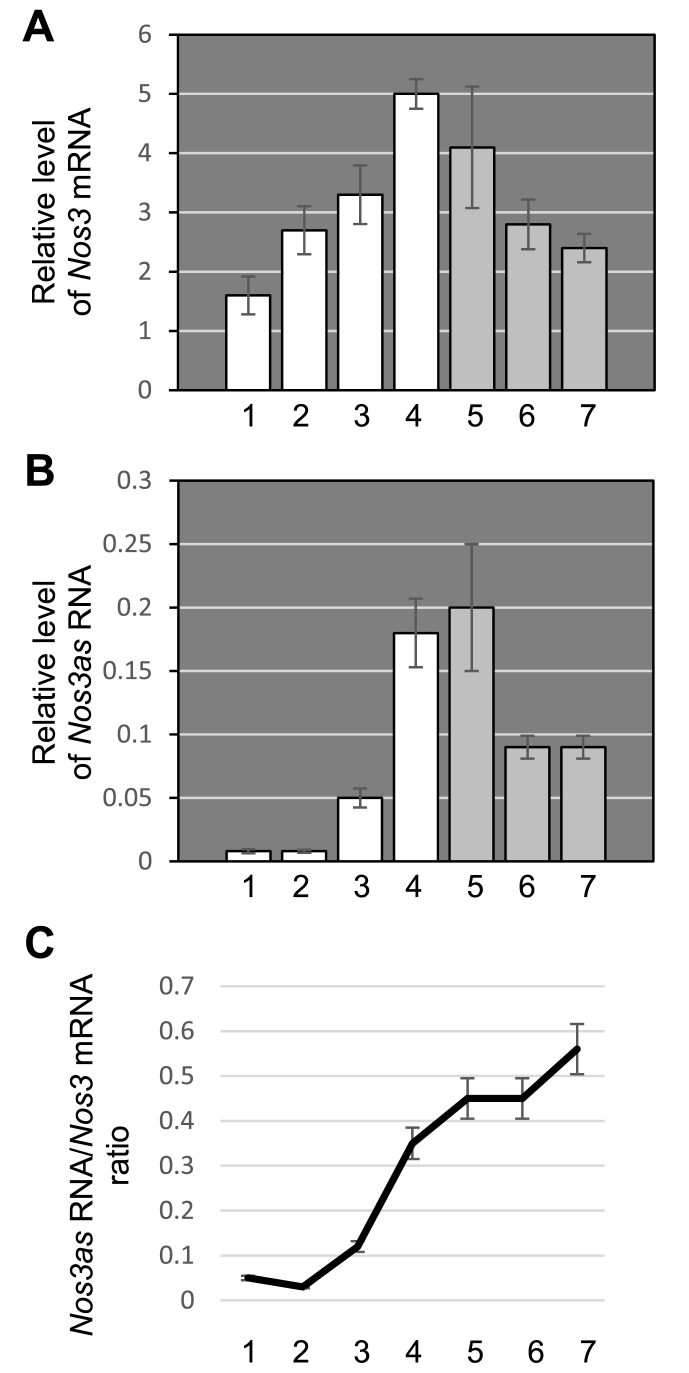
The expression profiles of *Nos3* mRNA and *Nos3as* RNA in the brain during embryonic development and postnatal life. (A) Quantitative RT-PCR analysis of *Nos3* mRNA expression. One-way ANOVA with post-hoc Tukey HSD test reveals that there is a statistically significant difference between 1 and 3 (p<0.01), 1 and 4 (p<0.01), 1 and 5 (p<0.01), 2 and 4 (p<0.01), 3 and 4 (p<0.01), 4 and 6 (p<0.01), 4 and 7 (p<0.01), 5 and 7 (p<0.05). There is no statistically significant difference between other pairs. (B) Quantitative RT-PCR analysis of *Nos3as* RNA expression. One-way ANOVA with post-hoc Tukey HSD test reveals that there is a statistically significant difference between 1 and 4 (p<0.01), 1 and 5 (p<0.01), 1 and 6 (p<0.05), 1 and 7 (p<0.05), 2 and 4 (p<0.01), 2 and 5 (p<0.01), 2 and 6 (p<0.05), 2 and 7 (p<0.05), 3 and 4 (p<0.01), 3 and 5 (p<0.01), 4 and 6 (p<0.05), 4 and 7 (p<0.05), 5 and 6 (p<0.01), 5 and 7 (p<0.01). There is no statistically significant difference between other pairs. Real-time RT-PCR experiments were performed on individual brains dissected at different stages of embryonic development (white bars) and postnatal life (grey bars). (C) The ratio between *Nos3as* RNA and *Nos3* mRNA expression. The ratios were calculated as 2^-ΔCt(*Nos3as*)^/2^-ΔCt(*Nos3*)^. 1 - E9.5, 2 - E11.5, 3 - E15.5, 4 - E18.5, 5 - P1, 6 - P20, 7 - 4 months.

Previous studies have shown that NO produced by Nos3 plays an important role in the regulation of neural precursor cell proliferation [14], [15]. Since *Nos3as* acts as a negative regulator of *Nos3* gene expression [5] it would be worthwhile to investigate if this NAT is also involved in neurogenesis. Consequently, we conducted a quantitative real-time RT-PCR analysis of *Nos3as* RNA and *Nos3* mRNA expression at different stages of mouse brain development including embryonic day (E) 9.5, E11.5, E15.5 and E18.5, postnatal day (P) 1 and P20, and 4-month-old adults. Our data show that *Nos3* mRNA expression undergoes a slow modest increase during embryogenesis but gradually decreases during the postnatal period (Fig. 1A). The temporal expression profile of the *Nos3as* RNA exhibits a noticeable difference. *Nos3as* RNA is present at a just detectable level in early embryonic brain but there is then a notable increase in its expression at E15.5. The *Nos3as* expression reaches its peak in newborn mice and rapidly decreases by P20 remaining quite stable until at least 4 months (Fig. 1B). These findings demonstrate that *Nos3as* RNA expression is differentially regulated during embryonic developments and in adults. Also, they indicate that the low level of *Nos3as* RNA coincides with phases of extensive neurogenesis in the embryo.

Another interesting observation is obtained when we determine the ratio between *Nos3as* RNA and *Nos3* mRNA expression (Fig. 1C). The calculations show that in the early embryonic stages (E9.5 and E11.5) the level of *Nos3as* RNA in the brain is very low compared to *Nos3* mRNA (approximately 1:20). This suggests that the reported suppressive effect of *Nos3as* RNA on the Nos3-NO pathway [5], [13] will be either very weak, or even negligible during these stages of development. However, at E15.5 the *Nos3as* RNA/*Nos3* mRNA ratio begins to grow and is further increased at E18.5 to approximately 1:3. The ratio remains relatively stable at later stages permitting an increase in the suppressive effect of *Nos3as* NAT on the *Nos3* expression.

### 3.2 Immunoblotting analysis shows an inverse correlation between the expression of *Nos3as* RNA and the level of Nos3 protein

**Fig. 2:**
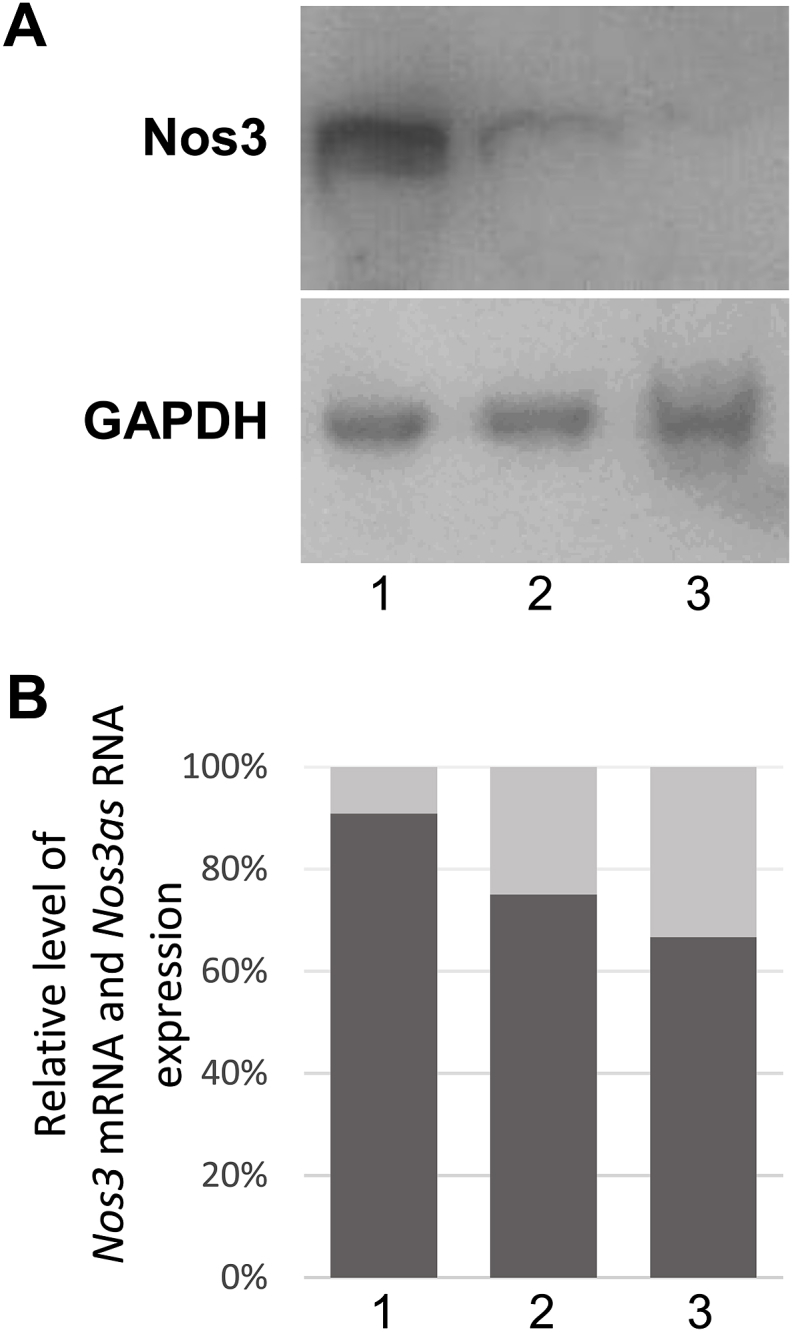
Inverse correlation between Nos3 protein production and relative expression level of *Nos3as* RNA. (A) A representative immunoblot showing the level of Nos3 protein in the mouse brain. Brain tissue lysates prepared from E15.5 (1), P5 (2) and 5 month old (3) mice were subjected to Western blot analysis with either anti-Nos3 (the upper section) or anti-GAPDH (the lower section) antibodies. (B) The 100% stacked column chart shows the relative percentage of *Nos3as* RNA (light grey) and *Nos3* mRNA (dark grey) expression levels in brain tissues dissected from E15.5 (1), P5 (2) and 5 month old (3) animals.

Our findings that *Nos3as* RNA is differentially expressed during embryonic and post-embryonic stages of brain development raised the possibility that this NAT could play a role in neurogenesis through the regulation of *Nos3* gene expression. To provide support for this hypothesis we investigated whether the observed changes in the relative expression level of *Nos3as* RNA are accompanied by alterations in Nos3 protein production. Specifically, we studied Nos3 protein expression in the embryonic (E15.5), early postnatal (P5) and adult (5 months) brain. The Western blot results demonstrate that Nos3 protein is present at a relatively high level at E15.5 but it is down-regulated at P5 and even further down-regulated in 5-month-old animals (Fig. 2A). Given similar levels of *Nos3* mRNAs in E15.5 embryos and in the early postnatal animals, these observations are indicative of post-transcriptional regulation of *Nos3* gene expression. In parallel experiments, we used brain tissue samples from the same animals for quantitative RNA analysis. As expected, we found that the *Nos3as* RNA/*Nos3* mRNA ratio is gradually increased over time after E15.5 (Fig. 2B). Thus, these observations reveal an inverse correlation between the relative expression of *Nos3as* RNA and the level of Nos3 protein produced in the brain.

### 3.3 Quantitative analysis of Nos3 mRNA and *Nos3as* RNA expression in the olfactory bulb

**Fig. 3:**
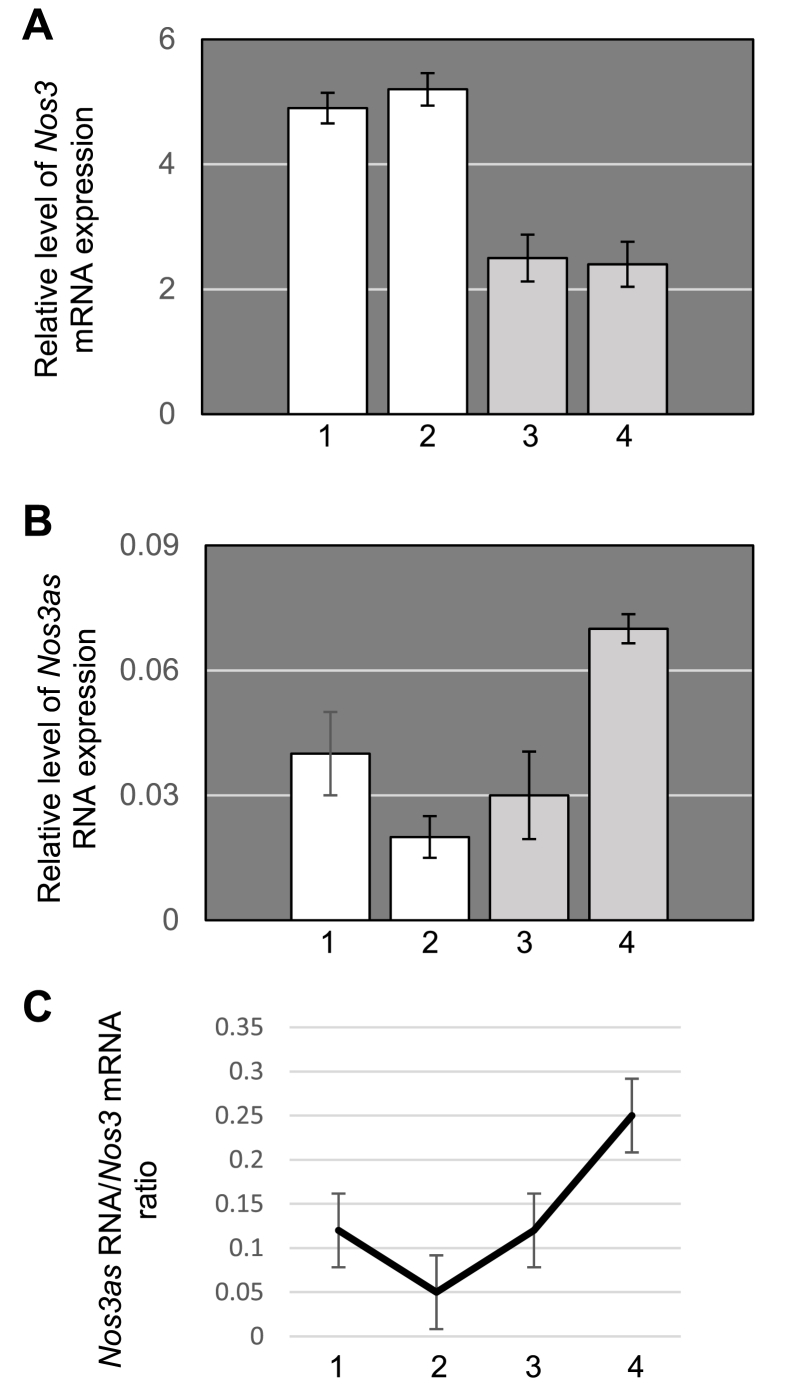
The expression profiles of *Nos3* mRNA and *Nos3as* RNA in the OBs during embryonic development and postnatal life. (A) Quantitative RT-PCR analysis of *Nos3* mRNA expression. One-way ANOVA with post-hoc Tukey HSD test reveals that there is a statistically significant difference between 1 and 3 (p<0.01), 1 and 4 (p<0.01), 2 and 3 (p<0.01), 2 and 4 (p<0.01). There is no statistically significant difference between other pairs. (B) Quantitative RT-PCR analysis of *Nos3as* RNA expression. One-way ANOVA with post-hoc Tukey HSD test reveals that there is a statistically significant difference between 1 and 4 (p<0.05), 2 and 4 (p<0.01), 3 and 4 (p<0.01). There is no statistically significant difference between other pairs. Real-time RT-PCR experiments were performed on OBs dissected from embryonic (white bars) and adult (grey bars) brain. (C) The ratio between *Nos3as* RNA and *Nos3* mRNA expression. The ratios were calculated as 2^-ΔCt(*Nos3as*)^/2^-ΔCt(*Nos3*)^. 1 – E15.5, 2 - E18.5, 3 – P24, 4 - 5 months.

It is known that neurogenesis in mammals mainly occurs during the embryonic development, but also in restricted regions of the adult brain. To examine if the observed association between active neurogenesis and down-regulation of *Nos3as* RNA is of importance, we performed quantitative RT-PCRs on RNA extracted from the olfactory bulb (OB). Notably, OB is a neural structure of the mammalian forebrain involved in adult neurogenesis. OBs dissected from the embryonic (E15.5 and E18.5), postnatal (P24), and adult (5 months) brain were used in this experiment. We found that the expression profile of *Nos3* mRNA in the OBs was generally similar to that observed at the level of the whole brain (Fig. 3A). The expression profile of *Nos3as* RNA, however, exhibited some recognisable differences. Firstly, *Nos3as* RNA is present at a very low level at E15.5, E18.5, and even at P24 (Fig. 3B). Secondly, the expression level of *Nos3as* RNA compared to that of *Nos3* mRNA remains very low up until P24 (Fig. 3C). Even in 5-month-old OBs this ratio is lower than those calculated for whole brains at E18.5, P1, P20 and 4 months. Taken together these findings suggest that the suppressive effect of *Nos3as* RNA on *Nos3* gene expression could be substantially delayed in the OB.

### 3.4 Nos3 mRNA and *Nos3as* RNA exhibit concurrent reciprocal changes in their expression during neuronal differentiation of Neuro2a cells

**Fig. 4:**
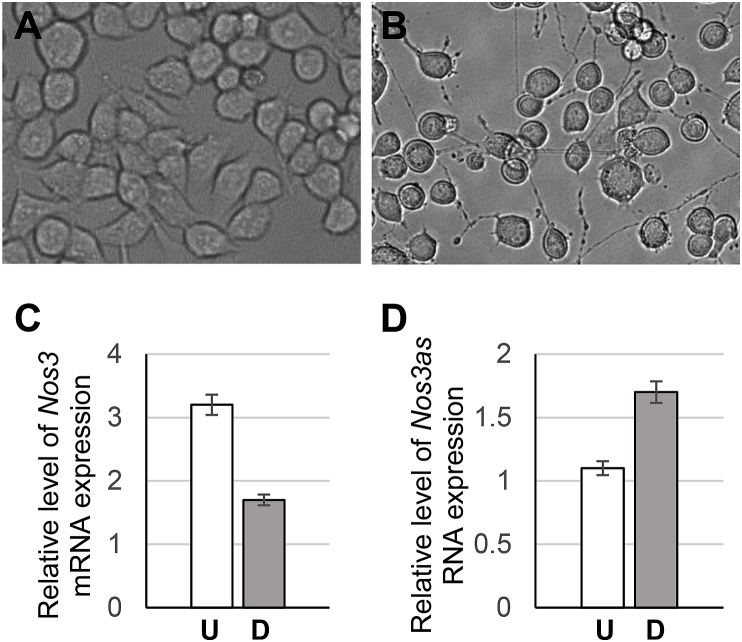
Quantitative analysis of *Nos3* mRNA and *Nos3as* RNA expression during neuronal differentiation of Neuro2a cells. (A) Undifferentiated Neuro2a cells. (B) Neuro2a cells differentiated into neurons. (C) Quantitative real-time RT-PCR analysis of *Nos3* mRNA expression. (D) Quantitative real-time RT-PCR analysis of *Nos3as* RNA expression. U – undifferentiated Neuro2a cells, D – differentiated Neuro2a cells. The values represent the average of three independent experiments.

Characteristic changes in the expression of *Nos3as* RNA during embryonic and post-embryonic stages of development of the brain and the OBs suggest a possible role of this NAT in the regulation of neurogenesis. To provide additional support for this hypothesis we chose the Neuro2a cell line, which is a well-established model system for studying the process of neuronal differentiation. We successfully induced the cells to differentiate into neurons by employing a combination of retinoic acid and reduced serum levels in the culture medium (Fig. 4A and B) and used them in our quantitative RT-PCR analysis. Interestingly, these experiments reveal concurrent reciprocal changes in the expression of the *Nos3as* RNA and *Nos3* mRNA in undifferentiated and differentiated Neuro2a cells. Specifically, the expression of the *Nos3* mRNA was higher in the undifferentiated cells than in the differentiated neurons (Fig. 4C), whereas the expression of *Nos3as* RNA exhibited the opposite dynamics, namely it was upregulated in differentiated neurons relative to the undifferentiated cells (Fig. 4D). These data indicate that *Nos3as* RNA could be involved in the regulation of neuronal differentiation of neuroblastoma cells through down-regulating the Nos3-NO pathway.

## 4 Discussion

Experimental work over the past several years has revealed an important role of NO generated by NOS enzymes in the regulation of neurogenesis. The common view among neuroscientists is that NO modulates the balance between proliferation and differentiation of neural stem cells in a concentration- and source-dependent manner [16], [17]. For example, it was demonstrated that NO produced by Nos3 stimulates proliferation of neural precursor cells [15], [18]. This finding was rather unexpected because Nos3 is traditionally believed to be primarily responsible for the production of NO within the cardiovascular system [19]. However, recent studies show that Nos3 plays an important role in physiological and pathophysiological processes in the brain such as synaptic plasticity, neuroprotection and neurogenesis [15], [18], [20], [21]. Notably, it is expressed in various brain cell types, including endothelial cells, astrocytes and several neuronal types [22], [23], [24].

Given the diversity of neural functions mediated by Nos3 and the well-known neurotoxic properties of excessive levels of NO [for a review, see 8], it is not surprising that Nos3 expression in the brain is tightly controlled. This is achieved through a variety of regulatory mechanisms operating at different levels [25], [26]. An additional important point emerges from recent studies showing that the expression of the Nos3-encoding gene in rodents and in humans is negatively controlled by *Nos3as* RNA [5], [13]. *Nos3as* RNA is a long *cis*-encoded NAT, which is also known as sONE or autophagy 9-like 2 RNA [27]. Because Nos3 was shown to be involved in the regulation of neural stem cell proliferation one may propose that *Nos3as* RNA is also an important part of the mechanisms regulating neurogenesis in mammals. However, while *Nos3as* RNAs was previously shown to be expressed in the brain, the question of its potential neuronal function has not been addressed yet.

Here we report the temporal expression profiles of *Nos3as* RNA and *Nos3* mRNA in the embryonic and adult brain. We found that the *Nos3as* RNA/*Nos3* mRNA ratio is very low in the early embryo but increases noticeably at later stages of embryogenesis and then remains quite high in the brain of adults. Since *Nos3as* RNA acts as a negative regulator of *Nos3* gene expression these data suggested that from E18.5 onwards the level of NO produced by Nos3 in the brain will be gradually decreased with age. Importantly, this proposition is supported by the results of our Western blot experiments, which revealed an inverse correlation between the relative expression level of *Nos3as* RNA and the level of Nos3 protein in the brain. Thus, considering the known proliferative role of Nos3-derived NO, these findings correlate well with published observations of high rate of proliferation of neural stem cells in the embryo and low rate of proliferation in the adult brain [for a review, see [28]]. Therefore, we can hypothesise that *Nos3as* RNA is involved in neurogenesis through the regulation of *Nos3* gene expression.

To provide additional support for our hypothesis we performed quantitative analysis of *Nos3as* RNA in the OBs. Our main reason for choosing OBs was that these structures of the brain are known to be active sites of the late stages of adult neurogenesis. Interestingly, our experiments have shown that the *Nos3as* RNA/*Nos3* mRNA ratio in the OBs remained very low in both embryonic and perinatal mice. This is in direct contrast to that observed at the whole-brain level. Consequently, we can speculate that the suppressive effect of *Nos3as* RNA on *Nos3* gene expression will be substantially delayed in the OBs allowing adult neurogenesis to occur.

Another interesting aspect of this study arises from the results of our experiments conducted on cultured marine Neuro2a cells. This cell line possesses the ability to differentiate into neurons in response to serum deprivation [29], [30]. Notably, Neuro2a cells have been successfully utilised to investigate the role of NO in neuronal differentiation [31]. Using this model system, we discovered that *Nos3as* RNA and *Nos3* mRNA exhibit concurrent reciprocal changes in their expression in undifferentiated and differentiated cells. Furthermore, we found that differentiation of Neuro2a cells in neurons is associated with up-regulation of *Nos3as* RNA. Thus, these *in vitro* observations indicate that the *Nos3as* RNA could be involved in the control of neuronal differentiation.

In the light of the data reported here we can suggest that the *Nos3as* RNA may play a role in neurogenesis through the regulation of the Nos3-NO pathway. Our findings, however, do not rule out other mechanisms by which this NAT can execute its function in the brain.

## Conflict of interest

The authors declare that there are no conflicts of interest.
